# Medicinal plants of South India: A comprehensive dataset for species identification

**DOI:** 10.1016/j.dib.2025.111660

**Published:** 2025-05-20

**Authors:** Muthukumar Arunachalam, T. Gopu, K. Uma, Sabari Nathan

**Affiliations:** a Department of Electronics Communication and Engineering, Kalasalingam Academy of Research and Education, Krishnankoil, Srivilliputhur, Tamil Nadu 626126, India; b Department of Computer Science and Technology, Sasi Institute of Technology & Engineering, West Godavari, Andhra Pradesh 534101, India; c AI Engineer, Couger Inc., Japan

**Keywords:** South Indian medicinal plants, Classification, Species recognition, Ethnomedicine

## Abstract

The identification and classification of medicinal plants are crucial for botanical research, traditional medicine, and AI-driven applications. However, the absence of a standardized, high-quality dataset limits advancements in automated species recognition. This study introduces SIMPD Version 1 (South Indian Medicinal Plants Dataset), a curated dataset comprising high-resolution images of diverse medicinal plant species native to South India. The dataset integrates detailed taxonomic classifications and metadata to facilitate precise species identification and biodiversity analysis. Images were acquired under real-world conditions, considering variations in illumination, pose, and environmental factors to enhance dataset robustness. SIMPD is designed to support machine learning applications, particularly in image-based plant classification, object detection, and segmentation tasks. By providing an extensive dataset for AI-driven research, this work aims to bridge the gap between traditional ethnobotanical knowledge and modern computational methodologies, fostering advancements in medicinal plant classification, conservation, and ecological research

Specifications Table

**Table:** 

Subject	Health sciences, Medical Sciences & Pharmacology
Specific subject area	Focuses on advanced computational techniques for identifying and analyzing plant species.
Type of data	Images
Data collection	The SIMPDVersion1 dataset comprises a meticulously categorized collection of high-quality images of medicinal plants native to specific regions in Tirunelveli, Tamil Nadu, South India. These images were manually captured using mobile cameras with varying resolutions, introducing significant diversity in pose, illumination, and resolution—enhancing the dataset's robustness for real-world applications. The dataset is thoughtfully curated to align with the traditional knowledge and practices of the local population, emphasizing the cultural and medicinal significance of these plants in South Indian daily life.To ensure high-resolution imagery, the dataset includes images captured in three distinct dimensions: • 1156 × 650 pixels (76 MP camera) • 2016 × 4480 pixels (74 MP camera) • 2084 × 4624 pixels (86 MP camera) The medicinal plants were predominantly found in natural habitats, such as areas near water tanks, lakes, and ponds, reflecting their ecological significance. To account for environmental variations, images were taken at different times of the day—morning (7:00–9:00 AM) and evening (4:00–6:00 PM)—under diverse lighting conditions. By capturing the unique ecological context of South Indian medicinal flora, this dataset serves as a valuable resource for species identification, classification, and AI-driven plant analysis, fostering advancements in botanical research, biodiversity conservation, and machine learning applications.
Data source location	City : Tirunelveli Country: India Latitude and Longitude for the collected samples/data: 8°45′16.2″N 77°43′32.3″E
Data accessibility	Repository name: Mendeley data Data identification number: 10.17632/9d89vjcghv.2 Direct URL to data: https://data.mendeley.com/datasets/9d89vjcghv/2
Related research article	None

## 1 Value of the Data

• **Comprehensive Dataset:** SIMPD Version 1 provides a systematically curated collection of high-resolution images of 20 medicinal plant species indigenous to South India, facilitating precise species identification and biodiversity analysis.
• **Enhancing AI Research:** The dataset supports the development and benchmarking of machine learning models for plant classification, object detection, and segmentation. The inclusion of diverse imaging conditions enhances model generalizability for real-world applications [[1], [2], [3]].
• **Bridging Traditional Knowledge and Computational Science**: SIMPD integrates ethnobotanical insights with computational methodologies, aiding researchers in preserving and digitizing traditional medicinal knowledge while enabling automated plant species recognition.
• **Application in Agriculture and Environmental Science:** Insights derived from SIMPD can be extended to related fields such as agricultural monitoring, forestry, and environmental conservation. Moreover, the dataset supports research on medicinal plants used in traditional practices like Siddha and Ayurveda, bridging the gap between traditional knowledge and modern scientific advancements.

## 2 Background

Medicinal plants play a crucial role in traditional healthcare systems and modern pharmacological research. South India, known for its rich biodiversity, is home to a vast number of medicinal plant species used in ethnomedicine, including Siddha, Ayurveda, and Unani practices. However, the accurate identification and classification of these species remain a challenge due to the absence of standardized, publicly available datasets that capture plant variations under real-world conditions.

Existing plant datasets are often developed in controlled environments with plain backgrounds, limiting their effectiveness for real-world applications. Furthermore, variations in plant morphology, growth stages, lighting conditions, and background clutter introduce significant challenges for automated classification models. Current datasets also lack adequate representation of indigenous medicinal plants specific to South India, making it difficult for researchers to develop AI-driven solutions tailored to this region’s flora.

To address these gaps, we introduce SIMPD Version 1, a curated collection of 2503 high-resolution images covering 20 diverse medicinal plant species. The dataset captures images in natural habitats, considering varying environmental conditions, poses, and illumination levels. It is designed to support AI-driven plant identification, machine learning-based classification, and conservation research. By providing a robust dataset, this work aims to enhance automated species recognition, aid in biodiversity conservation, and bridge the gap between traditional ethnomedicine and modern computational research.

## 3 Data Description

Automated plant recognition in real-time, wild environments remains a significant challenge in both botanical taxonomy and computer vision. Existing medicinal plant databases are often designed for specific plant classes and are typically captured in controlled laboratory settings with plain backgrounds, limiting their real-world applicability. Traditional handcrafted feature engineering struggles to process large-scale, unconstrained datasets, making it inadequate for modern AI-driven plant identification [4]. Moreover, existing datasets lack mobile-based plant images captured in natural scenes, leading to inconsistencies due to variations in contributors, camera types, geographic locations, seasonal changes, and individual plant characteristics.

To bridge this gap, we introduce SIMPD Version 1 (South Indian Medicinal Plants Dataset), a carefully curated collection of 2503 high-resolution images covering 20 medicinal plant species indigenous to South India. The dataset was collected in outdoor environments under natural lighting conditions, ensuring realistic variability in illumination, shadows, and reflections. To capture the ecological context of the plants, images were taken in their native habitats, including areas near water bodies (ponds, lakes, and irrigation canals), agricultural lands, and forest patches. The complete details of existing datasets are summarized in Table 1.

**Table 1:** Details of the existing plants databases.

Database Name	Country origin	No of plant species	Total number of Images	Memory size	References
Swedish leaf	Sweden	15 tree species	1125	3.705 GB	http://www.cvl.isy.liu.se/en/research/datasets/swedish-leaf/
Flavia Dataset	China	32 plants	1907	1 GB	http://flavia.sourceforge.net/
Image CLEF 11	France	71 tree species	6436	154 KB	http://www.imageclef.org/
Image CLEF 12	France	100 tree species	11,572	816 KB	http://www.imageclef.org/
Leaf Snap	United States	185 tree species	23147	977 MB	http://leafsnap.com/dataset/
Oxford Flower 17	United Kingdom	17 flower species	1360	218 MB	https://www.robots.ox.ac.uk/∼vgg/data/flowers/17/
Oxford Flower 102	United Kingdom	102 flower species	8189	329 MB	https://www.robots.ox.ac.uk/∼vgg/data/flowers/17/
ICL	China	220 plant species	17,032	-	http://www.intelengine.cn/English/dataset
MEW	Europe	153 species	9745	1.33 GB	http://zoi.utia.cas.cz/node/662
Malayakew	England	44 Plant species	2816	383 MB	https://web.fsktm.um.edu.my/∼cschan/downloads_MKLeaf_dataset.html

Several publicly available datasets, such as those on Mendeley, Kaggle, and other research repositories, focus on fruit and general plant classification. However, these datasets often have significant limitations when applied to medicinal plant identification, particularly in real-world, unconstrained environments. SIMPD Version 1 addresses these gaps and offers unique advantages:

### 3.1 Region-Specific Medicinal Plant Dataset

Most publicly available datasets focus on general plant or fruit species from diverse regions, but they lack datasets tailored specifically for South Indian medicinal plants. SIMPD Version 1 is designed to support ethnobotanical research and traditional medicinal knowledge preservation, making it highly valuable for researchers working on Siddha, Ayurveda, and Unani practices.

### 3.2 Real-World Environmental Variability

Many existing datasets, such as the Flavia Leaf Dataset or Swedish Leaf Dataset, are captured in laboratory environments with plain backgrounds. SIMPD captures images in natural settings, including agricultural lands, water bodies, and wild vegetation, which introduces realistic lighting conditions, occlusions, and varying angles—enhancing model generalization for real-world applications.

### 3.3 High-Resolution Images with Diverse Capturing Conditions

Most existing datasets contain images captured under uniform conditions with a single camera type and resolution. SIMPD Version 1 provides images from multiple cameras (76 MP, 74 MP, and 86 MP) with resolutions ranging from 1156 × 650 pixels to 2084 × 4624 pixels. Captured under morning and evening light, ensuring illumination diversity to support deep learning models with robust training samples.

### 3.4 Inclusion of Full Plant Structures, Not Just Leaves or Fruits

Existing datasets such as LeafSnap or Fruit360 (on Kaggle) focus on isolated leaves or fruits, which may not fully represent a plant’s taxonomy. SIMPD Version 1 includes entire plant structures, such as stems, flowers, and leaves, to support comprehensive species identification.

### 3.5 Ethnobotanical Significance and Traditional Knowledge Integration

Unlike standard fruit or plant datasets that focus only on classification, SIMPD integrates metadata on medicinal applications, ecological significance, and local names, making it valuable for biodiversity conservation, herbal medicine research, and sustainable agriculture studies. This ensures that the dataset not only aids AI-based classification but also serves as a resource for scientific and cultural knowledge preservation.

**Fig. 1:**
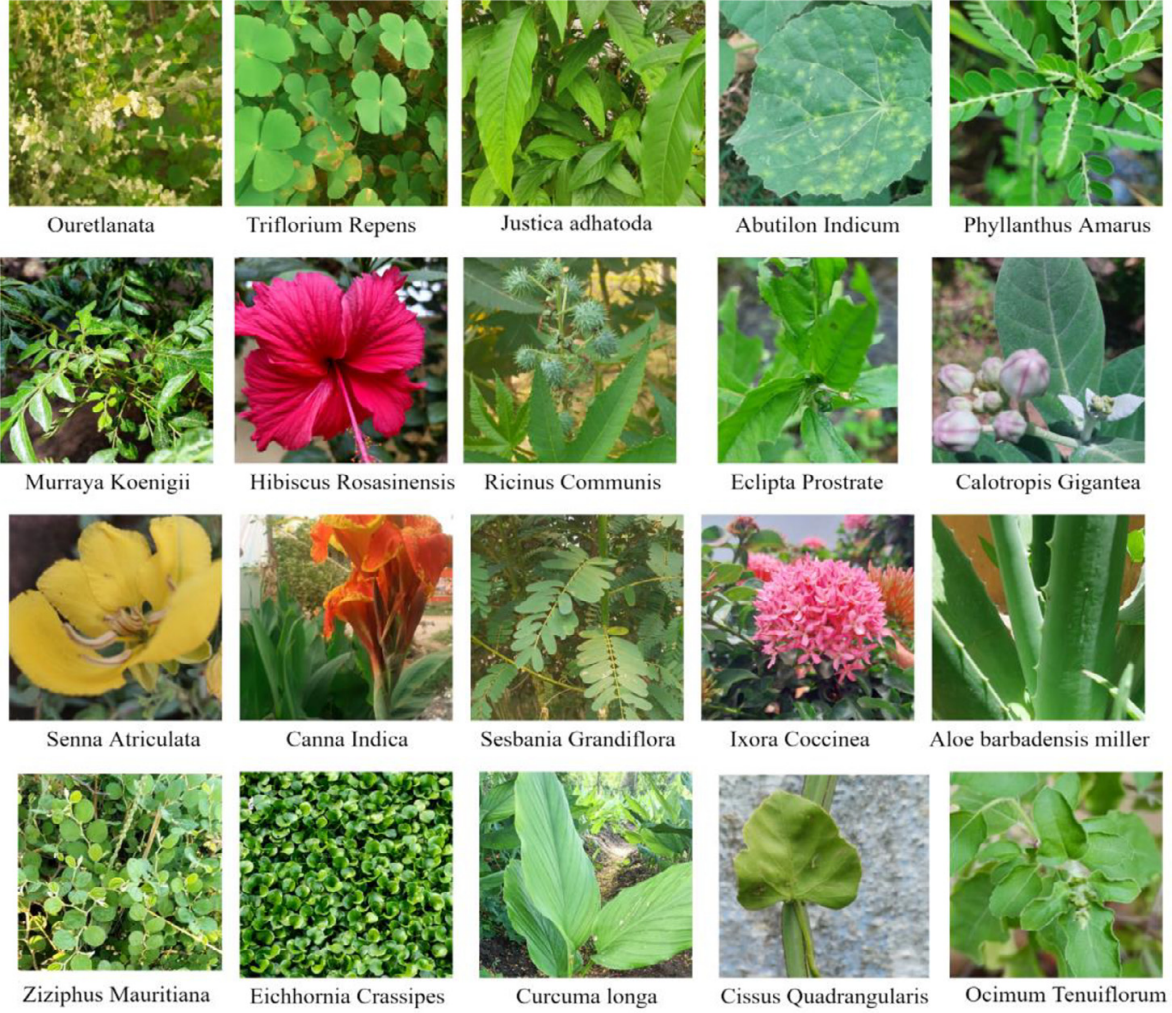
Sample images of SIMPD v1 shown in .jpg format.

Fig. 1 showcases all 20 different species and the details of the medicinal plants along with their colloquial name, Botanical name and the number of images are shown in Table 2.

**Table 2:** Details about each species in SIMPDv1.

Vernacular/Colloquial Name	Common Name	Species Category	Number of Images
Adathoda	MalabarNut	Justica adhatoda	100
Aagaya Thamarai	Water Hyacinth	Eichhornia Crassipes	192
Aamanaku	Castor Seeds	Ricinus communis	109
Aarangkeerai	European water clover	Trifolium Repens	100
Aavarai	Avaram senna	Senna Atriculata	125
Agathi	West India Pea	Sesbania grandiflora	91
Chembarathi	Hawaiian Hibiscus	Hibiscus Rosasinensis	113
Elandhai	Indian Jujibee	Ziziphus mauritiana	92
Erukan Elai	Crown flower	Calotropis gigantea	106
Thulasi	Mint		102
Thuthi	Indian Mallow	Abutilon Indicum	165
Idli Poo	Flame of woods	Ixora coccinea	114
KalVaazhai	Indian Shot	Canna indica	149
Kannupeelai	Mountainknot grass	Ouretlanata	119
Karisalankanni	Bhringaraj	Eclipta prostrate	208
Karuvepilai	Curry leaves	Murraya koenigii	118
Katralai	Aloe vera	Aloe barbadensis miller	145
Keelanelli	Stone Breaker	Phyllanthus amarus	111
Manjal	Turmeric	Curcuma longa	110
Pirandai	Veldt grape	Cissus quadrangularis	134

## 4 Challenges of the Dataset

### 4.1 Intra-Class Variability and Inter-Class Similarity

Plants with Similar Colored Flowers (Fig. a): Many species have flowers with identical colors but belong to different botanical categories. AI models trained solely on color-based features may misclassify them, requiring texture and shape-based feature extraction for differentiation.

Different Plants with Similar Leaves (Fig. d): Some plant species exhibit leaf structure similarities, making classification difficult. Deep learning models must rely on subtle vein patterns, margins, and surface texture rather than shape alone.

### 4.2 Environmental Variability Affecting Image Consistency

Lighting Variations (Fig. b): Images were taken under different illumination conditions, affecting contrast, shadow depth, and color accuracy. This may lead to inconsistent model predictions, requiring robust normalization techniques. Background Complexity and Clutter: Unlike laboratory-captured datasets with plain backgrounds, SIMPD images contain natural clutter, such as soil, surrounding vegetation, and overlapping plants, making it difficult for models to distinguish the primary plant from the background.

### 4.3 Species With Similar Structures But Different Attributes

Plants with Similar Leaves but Different Flowers (Fig. c): Some plants have almost identical leaf structures but exhibit different flower morphology. Models trained on leaf-based features may fail to distinguish such species unless flower characteristics are incorporated into the classification.

Different Plants with Similar Fruits (Fig. e): Certain medicinal plants produce fruits that resemble those of other species. Models relying on fruit-based classification must be trained with additional metadata (such as texture, size variations) to avoid confusion.

## 5 Experimental Design, Materials and Methods

**Fig. 2:**
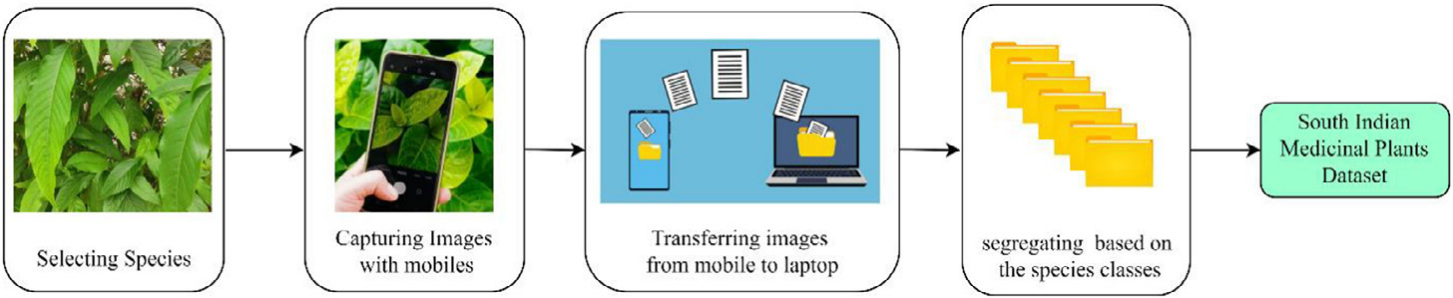
Process of data acquisition.

**Fig. 3:**
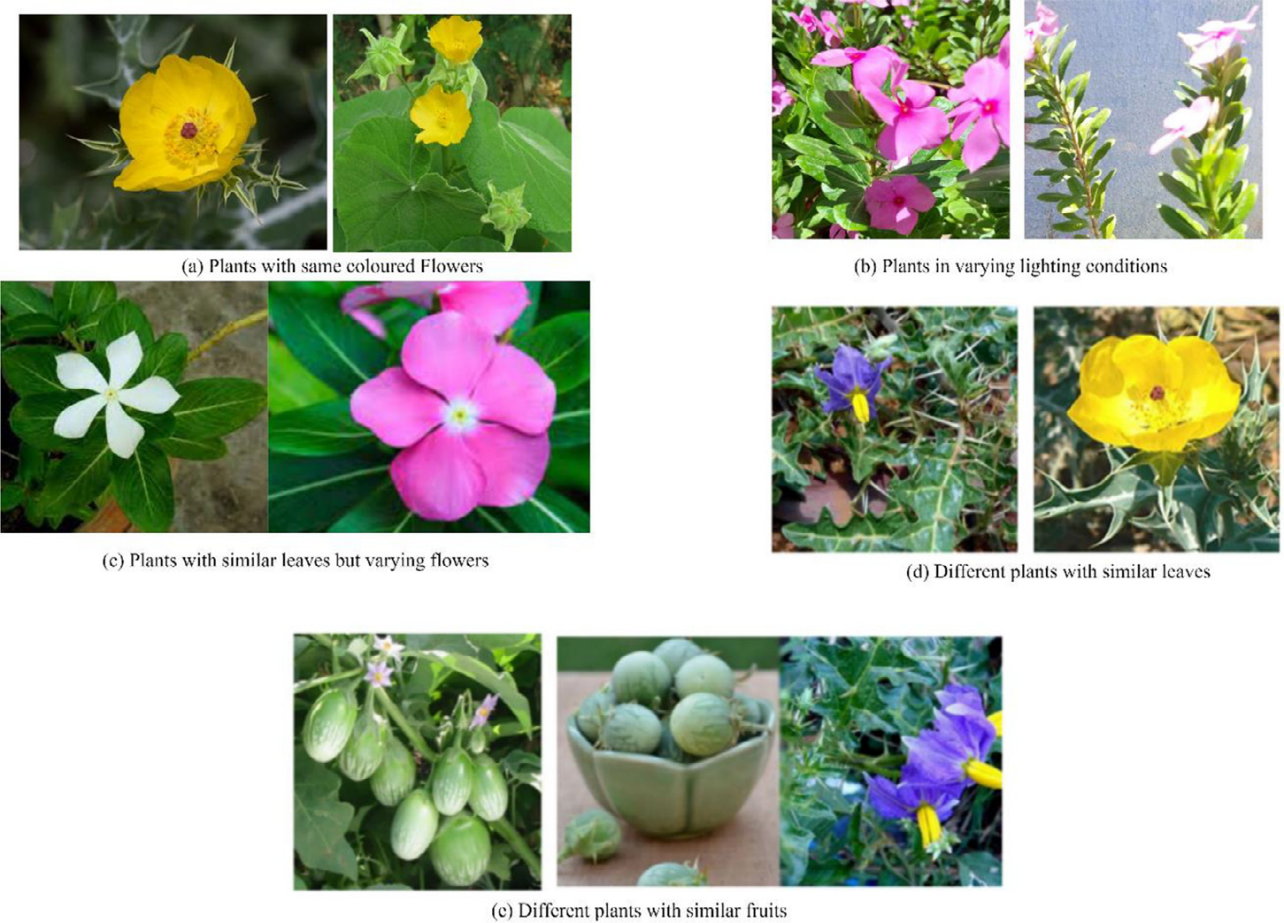
Similarities and variations in plants.

Plants are essential to human survival and play a vital role in maintaining Earth’s ecological balance by providing sustenance, shelter, and contributing to a breathable atmosphere. The SIMP dataset was carefully curated under unconstrained environmental conditions, capturing images with significant variations in illumination, scaling, view angles, as well as the growth stages and age of the plants. Additionally, the dataset features a broad spectrum of image variations, including differences in camera resolution, lighting conditions, and angles, ensuring a diverse and robust collection for real-world applications. The data acquisition process, as illustrated in Fig. 2, involved capturing images in the field and then transferring them to a laptop for subsequent analysis. After processing, the images were systematically categorized into 20 distinct plant classes (see Fig. 3), allowing for organized, efficient classification and facilitating advanced machine learning models for plant identification and research [5].

Based on this, a study focused on classifying six medicinal plant leaves using machine learning on a dataset collected from The Islamia University of Bahawalpur has been proposed. The multi-layer perceptron classifier achieved the highest accuracy of 99.01 %, outperforming models like random forest and logit-boost. The research optimized feature selection using a chi-square method and extracted multispectral and texture-based characteristics for classification [2]. An automated real-time plant species identification system for medicinal plants in Borneo, utilizing an EfficientNet-B1 deep learning model has also been developed. The system integrated a computer vision model, a knowledge base, and a mobile application, achieving up to 87 % accuracy on test datasets. It also incorporated crowdsourced feedback and geo-mapping, enhancing real-time plant identification capabilities [3]. A deep learning-based study employed a CNN model to classify six plant species in real-time using IoT, achieving 99 % accuracy. The CNN model outperformed traditional machine learning models, such as logistic regression and random forest. Future work aims to extend this research by automating plant growth estimation [5].

### 5.1 Selection of Medicinal Plants

The case study documents the indigenous knowledge of Kani tribal healers in the Tirunelveli Hills, who rely on medicinal plants as their primary source of healthcare. Ethnomedicinal data were collected through interviews over four years and analyzed using quantitative indices. The research highlights the traditional use of medicinal plants for treating common ailments and underscores the importance of preserving this knowledge [6]. An ethnobotanical survey conducted in Kancheepuram, Tamil Nadu, documented 85 medicinal plant species used by traditional healers for treating various diseases. The Euphorbiaceae family was found to be the most dominant, with leaves being the most commonly utilized plant part. The study emphasizes the risk of traditional knowledge disappearing, as fewer young people continue to practice these traditional healing methods [7]. A study on traditional medical practices and the use of 67 ethnomedicinal plant species by Kani tribal healers in Karayar, Tamil Nadu, was analyzed. The plants were used to treat 31 ailments, with cough being the most common (7 plants), and Fabaceae having the highest species count (10). Leaves of 23 species were primarily used, offering valuable insights for researchers and conservationists [8]. Another study documented the traditional medicinal knowledge of healers in Thimmarajapuram, Tirunelveli, focusing on the importance and use of medicinal flora. A total of 45 plant species from 27 families were recorded, with leaves being the most commonly used part, often prepared as juice or paste. Gastrointestinal ailments were the most frequently treated conditions [9].

Building on insights from previous case studies and the historical knowledge of local elders, we have carefully analyzed the medicinal significance of various plants and selected specific species to ensure accurate interpretations and reliable conclusions.

## Limitations

One major limitation of the dataset is its geographic concentration, as all images were collected from locations in and around Tirunelveli, South India, which may restrict its applicability to other regions. Variations in image quality, lighting conditions, and backgrounds introduce noise, posing challenges for automated recognition systems. Moreover, the dataset lacks detailed metadata, such as precise location coordinates, capture time, and plant health status, which could enhance contextual understanding. Addressing these limitations in future versions would significantly improve the dataset’s utility for broader applications in botanical research and computer vision

## Ethics Statement

The authors have reviewed and adhered to the ethical standards necessary for publication in Data in Brief. They affirm that the present study does not involve human subjects, animal experimentation, or the use of data obtained from social media platforms.

## CRediT authorship contribution statement

**Muthukumar Arunachalam:** Visualization, Investigation, Supervision. **T. Gopu:** Conceptualization, Methodology, Software, Validation. **K. Uma:** Data curation, Writing – original draft, Writing – review & editing. **Sabari Nathan:** Data curation, Writing – original draft, Software, Validation.

## Data Availability

Mendeley DataSIMPD V1: South Indian Medicinal Plants dataset (Version 1) (Original data).
